# Gut microbiota and its metabolites – molecular mechanisms and management strategies in diabetic kidney disease

**DOI:** 10.3389/fimmu.2023.1124704

**Published:** 2023-01-19

**Authors:** Zi-Hui Mao, Zhong-Xiuzi Gao, Dong-Wei Liu, Zhang-Suo Liu, Peng Wu

**Affiliations:** ^1^ Traditional Chinese Medicine Integrated Department of Nephrology, The First Affiliated Hospital of Zhengzhou University, Zhengzhou, China; ^2^ Institute of Nephrology, Zhengzhou University, Zhengzhou, China; ^3^ Henan Province Research Center for Kidney Disease, Zhengzhou, China; ^4^ Key Laboratory of Precision Diagnosis and Treatment for Chronic Kidney Disease in Henan Province, Zhengzhou, China

**Keywords:** gut microbiota, metabolite, diabetic kidney disease, immunity, therapy

## Abstract

Diabetic kidney disease (DKD) is one of the major microvascular complications of diabetes mellitus and is also one of the serious risk factors in cardiovascular events, end-stage renal disease, and mortality. DKD is associated with the diversified, compositional, and functional alterations of gut microbiota. The interaction between gut microbiota and host is mainly achieved through metabolites, which are small molecules produced by microbial metabolism from exogenous dietary substrates and endogenous host compounds. The gut microbiota plays a critical role in the pathogenesis of DKD by producing multitudinous metabolites. Nevertheless, detailed mechanisms of gut microbiota and its metabolites involved in the occurrence and development of DKD have not been completely elucidated. This review summarizes the specific classes of gut microbiota-derived metabolites, aims to explore the molecular mechanisms of gut microbiota in DKD pathophysiology and progression, recognizes biomarkers for the screening, diagnosis, and prognosis of DKD, as well as provides novel therapeutic strategies for DKD.

## Introduction

Diabetic kidney disease (DKD) is a pivotal complication of diabetes mellitus and significantly increases the risk of cardiovascular disease and end-stage renal disease (ESRD), that ultimately results in dialysis or high-mortality and economic burdens (1). The increased number of DKD and ESRD is partially attributed to lifestyle and dietary habits associated with diabetes and hypertension (2). Management and treatment strategy of patients with DKD includes controlling blood glucose, blood lipid, and blood pressure as well as blockade of the renin-angiotensin system (RAS); however, the risk of DKD still remains to be high (3) indicating the presence of unrecognized factors and mechanisms involved. The occurrence and progression of DKD is correlated to the interaction between gene and environment (4). Despite that hyperglycemia-induced metabolic alterations, hemodynamics changes, RAS activation, podocyte injury or loss, epithelial dysfunction, inflammation, and immunoreaction contributed to disease progression, specific molecular mechanisms and pathogenesis need to be explored (5).

The gut microbiota is powerful for maintaining host internal environmental homeostasis. For one thing, microbiome prevents infection caused by pathogens, promotes the digestion and absorption of nutrients, and synthesizes essential vitamins and amino acids (6). For another thing, it exerts an anti-inflammatory function (6), regulates fat metabolism (7), and participates in immune system development (8). And thirdly, gut microbiota-derived metabolites such as short-chain fatty acids (SCFAs), bile acids (BAs), lipopolysaccharide (LPS), and trimethylamine N-oxide (TMAO) are essential mediators of microbial-host crosstalk by interacting with host environment (9). The diversified, compositional, and functional alterations of gut microbiome are termed dysbiosis (10), which leads to a reduction in SCFAs and an increase in uremic toxins, activation of RAS, inflammation, and aggravated immune response. Nonetheless, specific mechanisms by which gut microbiota affects DKD have not been fully elucidated. This review summarized the role of gut microbiota and its metabolites in DKD, discussed underlying mechanisms of gut microbiota involved in DKD progression, and explored its potentials in DKD management and treatment.

## Gut microbiota and its metabolites

### Gut microbiota

**Figure 1:**
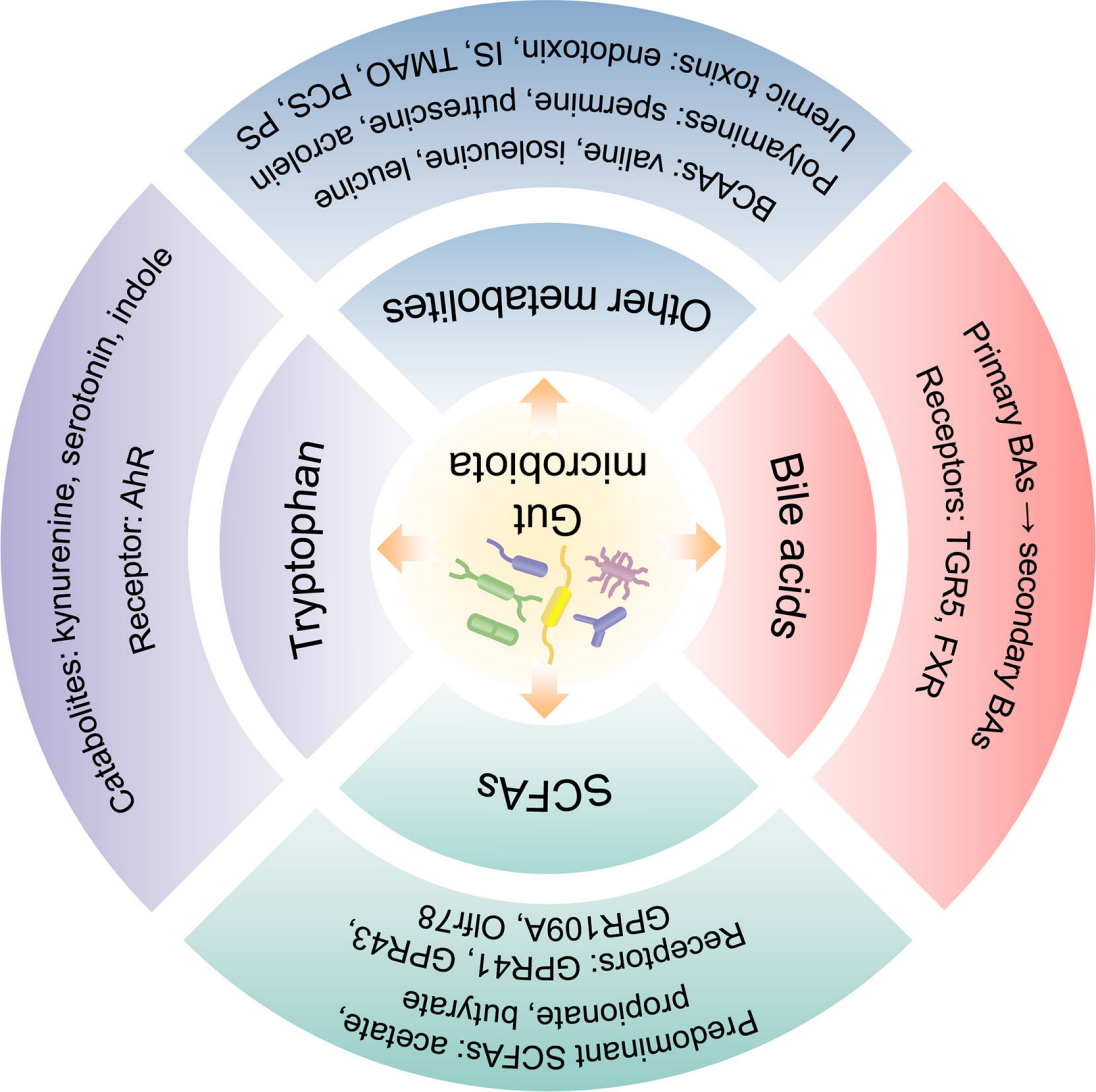
Gut microbiota-derived metabolites.

The human gastrointestinal tract possesses a plentiful microbial community which collects approximately 100 trillion microorganisms, including bacteria, fungi, viruses, phages, and archaea (11). Commonly, the gut microbiota is comprised of 6 phyla incorporating with *Bacteroidetes*, *Frimicutes*, *Verrucomicrobia*, *Proteobacteria*, *Actinobacteria*, and *Fusobacteria*, in which *Bacteroidetes* and *Frimicutes* are the majority components (12). The stability of intestinal microbiota is closely related to host health and disease. What is more, gut microbiota is symbiotic with the host and participates in a variety of physiological activities, such as fermenting food, resisting pathogens and regulating immune function (13). The gut microbiota contributes to host physiology by producing a multitude of metabolites (14) (**Figure 1**). Numerous metabolites derived from gut microbiota fermentation are vital factors in host-microbiota cross-talk and have been shown to be correlated with kidney function.

16S rDNA, metagenomics, and mass spectrometry can be utilized to explore the diversity, composition, and function of gut microbiota as well as microbiota-related serum metabolites in patients with DKD. Interaction studies between plasma metabolomics and gut microbiome in experimental DKD mouse/rat model provided evidence for the gut-metabolism-kidney axis, and verified the involvement of gut microbiota and circulating metabolites in DKD progression (15, 16). DKD patients displayed dysbiosis with composition, richness and diversity in gut microbiota (17–19). *Roseburia intestinalis* was significantly decreased while *Bacteroides stercoris* was increased in DKD patients (20). Furthermore, studies in early DKD caused by type 1 diabetes indicated that differences in gut microbiota and serum metabolite profiles were dependent on albuminuria levels (21). Several studies also revealed diversity and species differences in gut microbiota between DKD patients and non-DKD patients (22–24).

### SCFAs

SCFAs are produced by the fermentation of polysaccharides with the assistance of gut microbiota and are the main source of nutrition for colon epithelial cells. Acetate, propionate, and butyrate generated from the bacterial fermentation of dietary fiber are the predominant SCFAs (25). SCFAs have been shown to inhibit the activity of histone deacetylase (HDAC) and involve in G protein-coupled receptors (GPRs) mediated signaling pathway (26, 27). SCFAs can bind to GPRs such as GPR41, GPR43, GPR109A, and olfactory receptors (Olfr) 78, and then were absorbed into system circulation after reaching distant tissues. Furthermore, SCFAs were demonstrated to participate in the sustainment of intestinal barrier integrity (28), enhance glucose and lipid metabolism, restraint energy expenditure (29), and modulate immunoreaction and inflammatory responses (30). The reduction of SCFAs-producing bacteria as well as low serum and fecal SCFAs level may be correlated with kidney injury (31–33). Butyrate was reported to improve the intestinal barrier function by promoting the production of colonic mucin and tight junction proteins (ZO-1) (34). It could also mitigate oxidative stress, inflammation, and fibrosis in kidney disease through GPRs or HDAC (35–37). Serum valerate and caproate levels were negatively correlated with the progression of DKD to ESRD (38). It has been shown that acetate mediated the dysregulation of cholesterol homeostasis by activation of GPR43, thereby contributing to the tubulointerstitial injury of DKD (39).

### Bile acids

BAs are synthesized from cholesterol in the hepatocytes and participates in the absorption of lipid as well as metabolic or inflammatory signaling pathways (40). The primary BAs including chenodeoxycholic acid (CDCA) and cholic acid (CA), are indispensable for lipid and vitamin digestion and absorption by conjugating to glycine or taurine (41). Primary BAs could transform and decompose into secondary BAs *via* gut microbiota. The gut microbiota modulates BA metabolism process through deconjugation, dehydrogenation, and dihydroxylation of primary BAs (42). Additionally, the synthesis of BAs is influenced by cholesterol 7α-hydroxylase (CYP7A1) and sterol 27-hydroxylase (CYP27A1) regulating *via* gut microbiota (14). BAs are ligands for G protein-coupled bile acid receptor (TGR5) and nuclear hormone receptor farnesoid X receptor (FXR). Moreover, the profiles of BAs and gut microbiota influence each other. BAs could alter the composition of intestinal microbiota. Conversely, microbiota modulates the size and composition of the BA pool as well as BA signaling (43). BAs combine with TGR5 to improve insulin sensitivity *via* glucagon-like peptide-1 (GLP-1) and regulate energy expenditure in muscle or brown adipose tissue (44). The activation of FXR decreases lipogenesis and hepatic gluconeogenesis, and inhibits bacterial overgrowth and translocation by producing antimicrobial peptides (45). FXR and TGR5 play a renal protective role in diabetes and obesity-related kidney disease by regulating renal signaling pathways (46). Gentiopicroside inhibits the NF-κB signaling pathway *via* TGR5 activation, thereby alleviating inflammation and fibrosis in DKD (47).

### Tryptophan

An essential aromatic amino-acid, tryptophan, generally originates from daily diet such as fish, milk, oats, cheese. Besides the synthesis of proteins, dietary tryptophan could act as a precursor of critical metabolites including kynurenine, serotonin, indole, and its derivatives (48). Kynurenine, a tryptophan-derived metabolite produced by tryptophan 2,3-dioxygenase and indoleamine (2, 3)-dioxygenase, is correlated with kidney function (49, 50). Tryptophan is decomposed by bacterial tryptophanase into indole, which is a compound responsible for intercellular signal transduction, participating in the gene expression of intestinal epithelium connections and anti-inflammatory factors in intestinal epithelial cells, as well as maintaining host-microbiota homeostasis on the mucosa surface (51). As downstream critical metabolites, 3-(2-Hydroxyethyl) indole, 3-methylindole, and indoleacrylic acid were downregulated in the DKD model and were reinstated after treatment with Tangshen Formula (15). Some compounds produced by tryptophan metabolism are ligands for the aryl hydrocarbon receptor (AhR) and could induce AhR conformational changes. Moreover, these compounds are involved in the gene expression of pro-inflammatory factors, the metabolism of cytochrome P450 (CYP) superfamily CYP1A1, CYP1A2, CYP1B1 and cyclooxygenase-2 (COX-2), or the degradation of selective proteins (52). The deficient activation of AhR pathway could reduce the production of GLP-1 and interleukin (IL)-22, increase intestinal permeability and LPS translocation, which contribute to inflammation and insulin resistance (53). Based on the combined analysis of gut microbiota, serum metabolites and clinical indicators in DKD patients, phenylalanine and tryptophan metabolic pathways were demonstrated to be associated with the progression of DKD (54).

### Other metabolites

Branched-chain amino acids (BCAAs) are essential amino-acids synthesized by gut microbiota, including valine, isoleucine, and leucine. BCAAs modulate protein synthesis, glucose/lipid metabolism, insulin resistance, and immunity, as well as maintain homeostasis (55). Polyamines, such as spermine, putrescine, polyamine oxidase and acrolein, are participated in the development of kidney disease by altering the metabolism of intestinal microbiota (56). The dysbiosis of gut microbiota promotes the production of bacteria-derived uremic toxins, such as indoxyl sulfate (IS), endotoxin, TMAO, and p-cresyl sulfate (PCS), which increase intestinal permeability and transfer into the systemic circulation through the damaged intestinal barrier. Accumulation of uremic toxins in kidneys could lead to kidney dysfunction (57). TMAO, a gut microbiota-derived metabolite, was associated with mortality and renal outcome in type 1 diabetes (58). Higher serum TMAO levels increased the risk of abdominal aortic venture in hemodialysis patients (59). Phenyl sulfate (PS) contributed to podocyte damage and albuminuria and was shown to be related to the progression of DKD (60). Imidazole propionate, a metabolite produced by the breakdown of histidine *via* gut microbiota, was increased in type 2 diabetes, affecting host inflammation and metabolism (61). Both PS and TMAO could be involved in the development of DKD through a secretory associated senescence phenotype and chronic low-grade inflammation (62). IS and PCS contributed to the nephrology and cardiovascular toxicities *via* the activation of inflammation and oxidative stress (63). Additionally, several uremic toxins such as urea, TMAO, PCS, and 3-carboxylic acid 4-methyl-5-propyl-2-furan propionic (CMPF) were associated with glucose homeostasis abnormalities and diabetes incidence (64). The dysbiosis of Gram-negative bacteria and increased LPS level were detected in type 2 diabetes related DKD (65).

## Gut microbiota-related factors in DKD progression

### Insulin resistance

**Figure 2:**
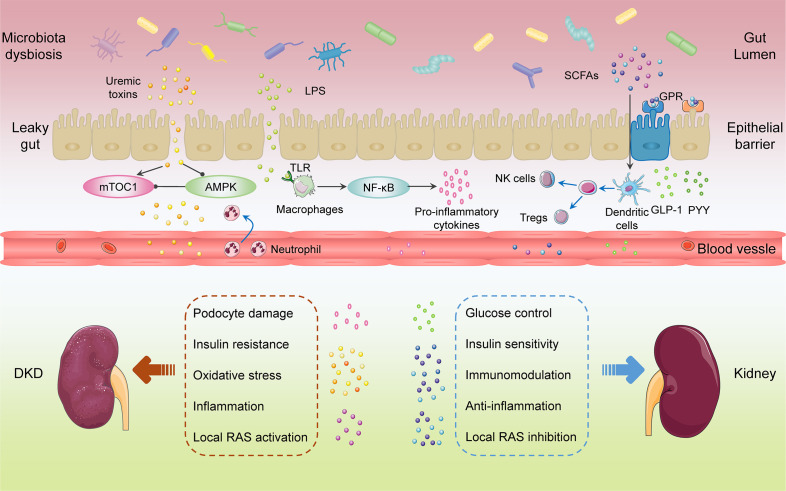
Gut microbiota-related factors in the progression of diabetic kidney disease.

DKD originates from metabolic dysregulation including hyperglycemia, hyperlipidemia, and insulin resistance (4). Hyperglycemia increases the generation of advanced glycation end products. The variance in insulin levels and insulin resistance might be a significant factor in DKD. Severe albuminuria and glomerulosclerosis were occurred in animals with complete deletion of podocyte insulin receptor (66). The dysbiosis of gut microbiota is linked to insulin resistance (67, 68) (**Figure 2**). A few species of microbiota, especially *Prevotella copri* and *Bacteroides vulgatus* are associated with insulin resistance and then impact host metabolism (69). Gut commensal *Bacteroides acidifaciens* could improve insulin sensitivity and may have therapeutic potential for diabetes and obesity (70). Microbiota depletion such as antibiotic-treated or germ-free mice could enhance insulin sensitivity and glucose tolerance (71). Podocyte insulin resistance caused podocyte injury and led to albuminuria in early DKD. Dysregulated GPR43 by gut microbiota dysbiosis resulted in podocyte insulin resistance through the inhibition of adenosine monophosphate-activated protein kinase (AMPK)-α activity (72). Butyrate enhanced AMPK phosphorylation and increased GLP-1 secretion, thereby alleviating insulin resistance and renal failure (34). Imidazole propionate, a microbial histidine-derived metabolite, may contribute to insulin resistance through activation of mechanistic target of rapamycin complex1 (mTORC1) (73).

### RAS

RAS is critical in the pathogenesis and progression of DKD. Moreover, local RAS might play a greater role than the circulating RAS (74). The secretion of renin in the juxtaglomerular apparatus plays an important role in the activation of intrarenal RAS by hyperglycemia. Olfr78 expressed in the renal juxtaglomerular afferent arteriole responded to signals from intestinal microbiota by mediating renin secretion, after that SCFAs could modulate blood pressure through Olfr78 and GPR41 (75). Succinate accumulated in the distal nephron-collecting duct, and activation of GPR91 responded to hyperglycemia through the stored (pro)renin and provoked tissue injury in DKD (76). The activation of intrarenal RAS by gut microbiota dysbiosis-derived excessive acetate was involved in the kidney injury of early DKD (77). Gut microbiota could promote angiotensin II (Ang II)-induced vascular dysfunction and hypertension by facilitating CCL2/IL-17-driven vascular immune cell infiltration and inflammation (78). Conversely, butyrate exerted an improvement for Ang II-induced renal injury and an antihypertension action by attenuating expression of (pro)renin receptor and renin as well as suppressing the (pro)renin receptor-mediated intrarenal RAS (79). During the fermentation of probiotics, angiotensin converting enzyme (ACE) inhibitory peptide and renin inhibitory peptide could be released, which are beneficial for lowering blood pressure (80, 81). In addition, ACE2 was associated with tryptophan metabolism and was sensitive to intestinal inflammation (82). A few uremic toxins such as IS and PCS are important stimulator of local RAS. Moreover, the inhibition of RAS ameliorated IS and PCS induced renal fibrosis (83).

### Inflammation

Inflammation accompanies the pathogenesis and progression of DKD whereas anti-inflammatory therapies might be beneficial for alleviating renal damage in DKD. Several inflammatory pathways participate in the complicated molecular networks and processes in DKD, including chemokines (CCL2, CX3CL1 and CCL5), inflammatory cytokines (IL-1, IL-6, IL-18), adhesion molecules, E-selectin, α-actinin 4, transcription factor nuclear factor-kappa B (NF-κB), and tumor necrosis factor (84). The initial stage of the inflammatory response to injury or metabolic dysfunction involves the release of proinflammatory mediators and the recruitment of leukocytes. Therefore, targeting inflammatory-resolution pathways might contribute to impede the progression of DKD (85). SCFAs could be involved in the modulation of pro-inflammatory and anti-inflammatory responses by inhibiting HDAC directly and binding GPRs indirectly (86). SCFAs produced by dietary fiber fermentation decreased the expression of inflammatory cytokines, chemokines, and fibrosis-promoting proteins in experimental DKD, thereby reducing albuminuria, glomerular hypertrophy, podocyte injury, and interstitial fibrosis. Moreover, this process required the involvement of GPR43 or GPR109A (87). Host/gut microbiota-derived tryptophan metabolites regulated AhR and then affected oxidative stress and inflammation in DKD (88). TMAO and PS accelerated kidney inflammation and fibrosis, resulting in development of DKD (60, 89). LPS, combined with toll-like receptors (TLRs) TLR2 and TLR4, participated in the inflammatory process of DKD through NF-κB activation and pro-inflammatory cytokines release, leading to the renal injury (90). Obesity enhanced intestinal permeability and chronic low-grade inflammation by inducing gut microbiota dysbiosis, ultimately causing the exasperation of DKD (91)

### Immunity

The activation of innate immunity through immune cells and resident renal cells contributed to the initiation and maintenance of inflammation (92). TLRs induced sterile tubulointerstitial inflammatory responses *via* NF-κB signaling pathway. The nucleotide-binding oligomerization domain, leucine-rich repeat and pyrin domain-containing 3 (NLRP3) inflammasome were associated with the connection of metabolic stress and pro-inflammatory cascades by inducing IL-1β and IL-18. The kallikrein-kinin system contributed to inflammatory progression by generating bradykinin and activating bradykinin receptors. Furthermore, coagulation enzymes promoted the activation of protease-activated receptors on kidney cells, leading to renal inflammation and fibrosis in DKD. Gut microbiota plays a significant role in maintaining host homeostasis as well as in modulating immune system (93). There have several studies characterizing the complex interaction between DKD, microbes and its metabolites, and immune responses. The microbiota colonized the intestinal tract after birth and regulated the antigenic responsiveness of lymphatic tissue (94). With the involvement of gut microbiota, the intestinal immune system started to build up and to be matured gradually. The dysbiosis of gut microbiota attracted immune cell activation and proinflammatory factors secretion, which led to immune dysregulation and inflammation (95). Mitochondrial antiviral signaling protein (MAVS), a component of innate immunity, was involved in maintaining intestinal integrity and barrier function. Damaged MAVS was conducive to the disrupted intestinal homeostasis, contributing to DKD progression (96). Microbiome-host interactions cooperatively maintained microbial community stability through metabolite-mediated innate immune modulation. What’s more, metabolites could influence the host’s immune homeostasis (97). Gut microbiota-derived metabolites passed through the intestinal barrier, accumulated in the circulation, recognized by immune system, and performed functions through gut-microbiome-immune axis (98). Bacteroids-derived SCFAs contributed to the activation of immune system by promoting neutrophil chemotaxis and inducing differentiation and proliferation of natural killer cells and Tregs (99).

## Management and treatment options for gut microbiota in DKD

### Clinical drugs

Various kinds of drug may alleviate DKD by affecting intestinal microbiota. Metformin was shown to contribute to several SCFAs-producing microbiota and increase the production of butyrate and propionate, thus participating in glucose homeostasis (100). Sodium-glucose cotransporter 2 inhibitor, as emerging antidiabetic drugs including empagliflozin, canagliflozin and dapagliflozin, restored the diversity of gut microbiota in experimental DKD mouse model. Moreover, reduced LPS production and increased SCFAs production by regulating the microbiota were observed in patients after inhibition of SGLT2 (101–103). Pirfenidone treatment increased gut microbial diversity in diabetic mouse model and reversed gut microbial dysbiosis and diabetic ketoacidosis biomarkers (104). Magnesium lithospermate B was found to ameliorate kidney injury by modulating gut microbiome dysbiosis and BAs metabolism (105). Abundant polysaccharides are beneficial for DKD. Polysaccharide from Armillariella tabescens mycelia, Cordyceps cicadae polysaccharide, and Bupleurum polysaccharide were demonstrated to modulate gut microbiota dysbiosis and inflammatory response (106–108). Traditional Chinese medicine such as Zicuiyin (109), Moutan Cortex polysaccharide (110), QiDiTangShen granules (111), Shenyan Kangfu tablet (112), and Tangshen Formula (113), have been used clinically to treat DKD. They had a significant curative role in regulating gut microbiota, eliminating intestinal toxins, inhibiting renal inflammation and immunity, alleviating renal injury, and protecting kidney function.

### Dietary intervention

**Figure 3:**
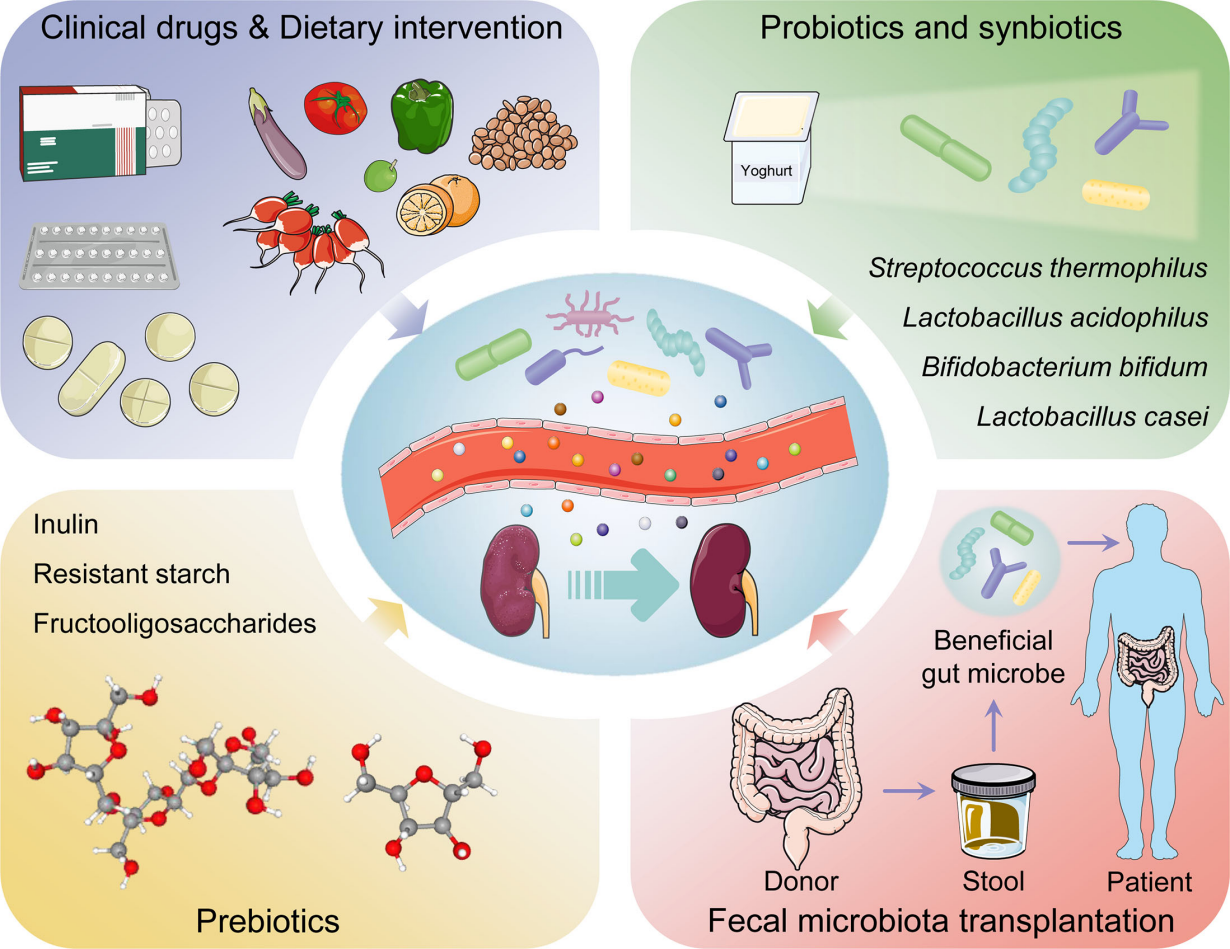
The management and therapeutic strategies of diabetic kidney disease based on gut microbiota.

Diet is fundamental to support human growth, health, and reproduction. Furthermore, diet was also shown to modulate and maintain the symbiotic gut microbiota communities colonized the intestinal tract (114). Under multiple host-containing endogenous and exogenous factors, diet becomes a pivotal determinant of the structure and function in gut microbiota (115) (**Figure 3**). The latest review regarding the effect of dietary nutrient intake on gut microbiota indicated that diet-microbiota crosstalk and personalized nutrition strategies are associated with chronic kidney disease progression (116). Moreover, the variation in dietary protein sources affected the gut microbiota, microbiota-derived metabolites, immune cell activation, and production of inflammatory cytokines (117). Studies from human population with different diets showed that *Bacteroides* was enriched in a protein-rich diets while *Prevotella* was enriched in a carbohydrate-based diets (118). Whole-plant fibers from fresh vegetables contained a lot of necessary micronutrients compared with highly processed fibers or fibers from seed coats (119). Plant-based low-protein diets seemingly contributed to postpone kidney replacement therapy by disturbing RAS, reducing proteinuria, and decreasing insulin resistance (120). Fermented and germinated foxtail millet whole grain diet raised the bacterial diversity especially probiotics, thereby ameliorating kidney injury in experimental DKD mouse model through inhibition of inflammation and immunity signaling pathways (121). A high linolenic acid diet aggravated gut microbiota dysbiosis and inflammatory responses in diabetes mouse model. Conversely, a low n-6/n-3 ratio diet improved glucose homeostasis, inhibited systematic inflammation, and ameliorated DKD (122). Punicalagin from pomegranates, a prospective bioactive polyphenol, was shown to alleviate diabetic kidney injury through gut-kidney axis (123).

### Probiotics and synbiotics

Probiotics contain live microorganisms that can change composition of microbiota and are supposed to provide health benefits to host (124). Synbiotics, a mixture comprising live microorganisms and substrates selectively utilized by host microorganisms confer a health benefit on the host (125) (**Table 1**). Probiotic and synbiotic supplementation, such as *Lactobacillus acidophilus*, *Lactobacillus casei* and *Bifidobacterium bifidum*, had beneficial effects on blood glucose and intestinal imbalance, production of uremic toxins, and inflammation or oxidative stress in diabetic hemodialysis patients (126–128). Furthermore, probiotics could ameliorate insulin resistance, stabilize fasting blood glucose levels, and improve antioxidant status (90, 129). Addition of probiotics such as *Lactobacillus acidophilus*, *Streptococcus thermophilus* and *Bifidobacterium longum* reduced the blood urea nitrogen level and uric acid concentration in patients with stage 3 and stage 4 chronic kidney disease (130, 131). Systematic review and meta-analysis demonstrated that probiotics might ameliorate high sensitivity-C reactive protein and oxidative stress biomarkers, as well as regulate lipid profile and anthropometric indices in DKD patients (132, 133).

**Table 1:** Differences between probiotics, synbiotics, prebiotics, and postbiotics.

Classification	Probiotics	Synbiotics	Prebiotics	Postbiotics
Definition	Live microorganisms that, when administered in adequate amounts, confer a health benefit on the host	A mixture comprising live microorganisms and substrate(s) selectively utilized by host microorganisms that confers a health benefit on the host	A substrate that is selectively utilized by host microorganisms conferring a health benefit	Preparation of inanimate microorganisms and/or their components that confers a health benefit on the host
Category	*Bifidobacterium* (*adolescentis*, *animalis*, *bifidum*, *breve* and *longum*); *Lactobacillus* (*acidophilus*, *casei*, *fermentum*, *gasseri*, *johnsonii*, *paracasei*, *plantarum*, *rhamnosus* and *salivarius*)	Complementary (prebiotic + probiotic); Synergistic (live microorganism + substrate)	Conjugated linoleic acids and polyunsaturated fatty acids; Oligosaccharides; Human milk oligosaccharides; Phenolics and phytochemicals; Readily fermentable	Inactivated strain (such as *Bacteroides xylanisolvens*, *Apilactobacillus kunkeei* and *Saccharomyces boulardii*); Bacterial lysates; Spirulina formulations
Health benefit	Healthy digestive tract construction (such as infectious diarrhoea, antibiotic-associated diarrhoea, and ulcerative colitis); Healthy immune system construction (including preventing allergic disease, decreasing inflammation, and enhancing anti-infection activities)	Treatment of NAFLD, obesity and metabolic syndrome, T2DM and glycaemia, IBS, CKD, dyslipidaemia, PCOS, AD, and inflammation; Prevention of surgical infections and complications, sepsis in infants, and AD; Eradication of *Helicobacter pylori*	Metabolic health; Satiety; Improved absorption of calcium and other minerals, bone health; Skin health; Digestive tract health; Allergy; Constipation; Immune function in elderly individuals	New antimicrobials; Targeted anti-inflammatory, immunoregulatory, and enhance vaccination efficacy agents; Novel signaling molecules that affect gut pain, sensation, secretion, and motility; Fermented infant formulas and bacterial lysates
Mechanism	Colonization resistance; Normalization of perturbed microbiota; SCFA production; Increased turnover of enterocytes; Regulation of intestinal transit; Competitive exclusion of pathogen; Vitamin synthesis; Bile salt metabolism; Gut barrier reinforcement	Complementary approach combines prebiotic (targets autochthonous beneficial microorganisms) and probiotic; Synergistic approach selects substrate that is utilized by the co-administered live microorganism, enhancing its functionality	Modulation of SCFA production; Promotion of beneficial microbiota; Bile salt metabolism; Alteration of bacterial growth and interaction with immune system; Enhanced secretion of satiety hormones peptide YY and GLP-1; Immunological modulation	Modulation of resident microbiota, immune responses, and systemic metabolic responses; Enhancement of epithelial barrier functions; Regulation of systemic signaling *via* the nervous system

NAFLD, non-alcoholic fatty liver disease; T2DM, type 2 diabetes mellitus; IBS, irritable bowel syndrome; CKD, chronic kidney disease; PCOS, polycystic ovarian syndrome; AD, atopic dermatitis; SCFA, short-chain fatty acid; GLP-1, glucagon-like peptide1.

### Prebiotics and postbiotics

Prebiotics such as noncarbohydrate food components, are substrates that are selectively used by host microorganisms for health benefits (134). The supplementation of prebiotics in daily dietary could exterminate pathogens, facilitate the growth of beneficial microorganisms, and regulate host intestinal microbiota (135). Moreover, prebiotic supplements might increase SCFAs levels (notably butyrate), restore intestinal barrier function, and relieve inflammatory response (136). Fructooligosaccharides could alleviate pathological changes in diabetes related kidney disease (137). Inulin-type fructans, a type of dietary fiber, was demonstrated to improve kidney diseases *via* modulating gut microbiota and SCFAs profile (138). Additionally, inulin-type fructans also decreased insulin resistance, serum insulin and fasting blood glucose levels, and increased fasting serum GLP-1 level in diabetes rats (139, 140). Resistant starch is a prebiotic compound that accelerates proliferation of health-promoting gut microbiota such as *Bifidobacteria* and *Lactobacilli*, increases the production of SCFAs, decreases the concentrations of uremic toxins and alleviates renal dysfunction (141). Postbiotics, defined as “preparation of inanimate microorganisms and/or their components that confers a health benefit on the host” in 2019 (142), have appeared increasingly in the literature and products; however, their effects on DKD are insufficient in research. Postbiotics exert immunomodulatory and intestinal barrier protective roles by increasing anti-inflammatory cytokine secretion and ZO-1 expression (143). Postbiotic-GABA-salt, spirulina formulations, sonicated *Lactobacillus paracasei* and *O. formigenes lysates* contribute to improve renal outcomes (144, 145).

### Fecal microbiota transplantation

Fecal microbiota transplantation (FMT) is a treatment in which the microbial community from a healthy donor’s stool was minimally transplanted into the patient’s intestinal tract (146). FMT is implemented with the purpose of restoring normal function of the gut microbiota and has generally been adapted into treatment for *Clostridium difficile* infection (147). Faecal microbiota is separated cautiously from selected donor’s stool, quantified in accordance with viable bacteria, and cryopreservation (148). Transplantable materials can be delivered in the form of encapsulated oral medication (149). As a true organ, gut microbiota is indispensable to human pathophysiology, suggesting that FMT might be an advantageous treatment for problems with metabolism, autoimmunity, and system development (150). Body weight gain, insulin resistance, albuminuria, and tumor necrosis factor-α levels in experimental DKD mouse model could be prevented by FMT (151). After six weeks post-FMT using stool derived from lean donors, the peripheral insulin sensitivity was significantly improved in male patients with metabolic syndrome, although the result was not sustained in following few weeks (152). Another double blind randomized controlled trial demonstrated that TMAO or proxies of vascular inflammation was undifferentiated in patients with metabolic syndrome received FMT from either lean donors or autologous (153). Hence, abundant experiments are needed to explore these potential therapeutic indications.

## Conclusion and perspective

The pathogenesis and pathophysiology of DKD incorporate not only hyperglycemia-induced metabolic alterations, hemodynamics changes, RAS activation, podocyte injury or loss, epithelial dysfunction, inflammation, and immune dysregulation, but also the influences of environmental factors and interactions between host and gut microbiota as well as its metabolites. Gut microbiota is associated with kidney disease, confirming the presence of gut-kidney axis through the involvement of genetic, immunity and dietary approaches. The gut microbiota participates in host homeostasis by producing a myriad of metabolites, which act ss key signaling molecules and substrates for metabolic reactions. The combination of metagenomics and metabolomics could help to investigate the relationship between dysbiosis of gut microbiota and metabolic disorders. Nonetheless, there are still complexities to overcome in identifying the potential causality of some metabolites from fully microbiota-derived or diet and host itself. High-quality microbiome analysis workflow is important to obtain reliable and repeatable results (154).

Dietary intervention, probiotics, synbiotics, and prebiotics are widely acceptable to patients in relative safety and traditional concept. However, various intestinal bacteria and metabolites have heterogeneous effects on host, some of which are beneficial to human health and others contribute to pathophysiology of diseases. Hence, it is necessary to investigate the signals and effects mediated by different bacteria and metabolites as well as reasonable application of bacteria community in the treatment strategies. Gut Microbiota-derived metabolites could act as biomarkers of DKD. Identification of biomarkers for screening, diagnosis, and prognosis of DKD as well as exploration of molecular mechanisms or pathways involved in DKD can facilitate individualized prevention and treatment. However, further studies involving human trials are needed to investigate the beneficial role of prebiotics, probiotics, synbiotics or FMT in DKD management by regulating gut microbiota. The therapeutic strategy targeting intestinal microbiota has prodigious potential in the future and will open an emerging perspective and orientation for DKD treatment.

## Author contributions

PW and Z-SL conceived the idea. Z-HM prepared the figures and tables, and drafted the manuscript. PW, Z-XG, D-WL and Z-SL revised the manuscript. All authors contributed to the article and approved the submitted version.
